# Health trends among workers in Germany and the role of changing job activities and working conditions

**DOI:** 10.1038/s41598-025-25692-z

**Published:** 2025-11-12

**Authors:** Johannes Beller, Julia Graßhoff, Batoul Safieddine, Stefanie Sperlich

**Affiliations:** 1 HMU Health and Medical University, Campus Düsseldorf, Kaistraße 16-16a, 40221 Düsseldorf, Germany; 2 Medical Sociology Unit, Hannover Medical School, Hannover, Germany

**Keywords:** Self-rated health, Workers, Job activities, Working conditions, Time trends, Epidemiology, Risk factors

## Abstract

Previous studies have found some evidence for worsening health trends in working age adults. This study aims to further investigate the time trends in self-rated health among workers and explore the potential role of changes in job activities and working conditions in explaining these trends. Data from the BIBB/BAuA Employment Surveys conducted in 2006, 2012, and 2018 were analyzed (N = 53,747, age 15+). The study variables included self-rated health as the dependent variable, and time period, age, gender, education, working hours, physical work activities, cognitive work activities, ergonomic working conditions, environmental working conditions, work intensity, work control, and work support as predictors. Logistic regression and mediation analyses were employed to study the associations between these variables and self-rated health over time periods. The findings revealed a significant deterioration in self-rated health among workers over the study period, alongside an aging and more educated workforce. Additionally, several working conditions and work activities underwent changes, with work becoming generally less physically demanding and more cognitively and psychosocially demanding. The changes in job activities and working conditions partly explained the negative trends in self-rated health, with work control and environmental conditions being most important. In conclusion, worsening trends in self-rated health among the working population were found. While changes in the world of work (especially perceived work control and hazardous environmental conditions) contribute to these trends, they constitute only part of the explanation. Further research is needed to identify further intermediary determinants driving these trends and develop targeted interventions to promote worker health and well-being.

## Introduction

The world of work is changing, driven by trends such as tertiarization, digitalization, educational expansion and the rise of the knowledge economy^1^. For example, tertiarization refers to the shift from agriculture and manufacturing towards service-oriented industries, which now account for a majority of occupations in many countries^2^. Alongside these changes, occupational safety measures have advanced, potentially considerably changing occupational health, working activities and working conditions^3^.

To understand how these changes in the world of work affect worker health, the Job Demands-Resources (JD-R) model provides a comprehensive theoretical framework^4^. The JD-R model posits that both job demands (such as physical, psychological, or organizational aspects including work intensity and environmental hazards) and job resources (such as aspects that help achieve job goals and reduce job demands, including work control and social support) influence worker health. In Germany’s rapidly changing work environment, which is characterized by demographic aging, educational expansion, tertiarization, and digitalization, traditional physical demands may have decreased while new psychosocial demands have emerged^5^. These social changes may have fundamentally altered the balance of job demands and resources, potentially creating new health challenges despite apparent improvements in classically hazardous working conditions.

Therefore, the changing world of work has immense implications for workers’ health. Research has shown that both work activities and working conditions can have a profound impact on the health of workers^6^. For example, physically or cognitive-emotionally intensive work activities have been found to be associated with the development of various health conditions^7,8^. Furthermore, it is well-known that physical and psychosocial working conditions can strongly influence workers’ health^9–11^, including the ergonomic conditions (e.g., lifting heavy loads), environmental conditions (e.g., working under loud noise), work intensity (e.g., working at a high pace), work control (e.g., having control about one’s work tasks), and work support (e.g., being supported by one’s colleagues) under which work is performed^12,13^. Given the significant impact of work activities and working conditions on the health of the working population and the changing world of work, it is essential to monitor health trends of the working population.

Several previous studies have investigated trends in the health of the working populations, with mostly concerning results. For example, a study by Ekblom-Bak and colleagues examined trends in cardiorespiratory fitness in the Swedish working force between 1995 and 2017^14^. This study, which included 354,277 participants aged 18–74 years, found a significant decrease in both absolute and relative fitness over the 22-year period. The proportion of individuals with low cardiorespiratory fitness nearly doubled, highlighting the potential impact of the trend. In another study, Beller and colleagues analyzed data from the Survey of Health, Ageing and Retirement in Europe (SHARE) study in Germany from 2004 to 2014^15^. They found that while employment rates generally increased over time, rates of disability also increased among adults aged 50–54, particularly for movement-related and activity-related limitations. The researchers predict that future workers will spend more of their working years with disabilities. This is because younger generations, who have more health limitations, will eventually replace the current older workforce who are generally healthier. As a last example, a study by Clause-Verdreau and colleagues examined health-related quality of life (HRQoL) in France from 1995 to 2016^16^. This study utilized repeated population-based cross-sectional surveys and found a substantial decrease in HRQoL scores across almost all subgroups, including employed individuals.

While such studies have provided valuable insights, more research is needed to better understand the time trends in workers’ health. Building on the JD-R model and the broader context of social change in Germany, we hypothesize that self-rated health among German workers has deteriorated over time. Specifically, we expect that despite improvements in traditional physical working conditions, the overall health impact has been negative due to increases in psychosocial job demands (such as cognitive complexity and work intensity) and decreases in job resources (such as work control and social support). We further hypothesize that changes in job demands and resources will partially mediate the relationship between time period and self-rated health, with different work characteristics contributing differentially to the observed health trends. The current study aims to help close this gap in the literature by investigating time trends in self-rated health among workers and determining whether these trends can be explained by the changing world of work as operationalized by changing work activities and working conditions over time. We ask: "How has self-rated health among workers changed, and can these trends be explained by changing work characteristics?".

## Methods

### Sample

Data were drawn from the Employment Surveys of the German Federal Institute for Vocational Education and Training (BIBB) and the German Federal Institute for Occupational Safety and Health (BAuA) conducted in 2006, 2012, and 2018. This study thus uses a repeated cross-sectional design, analyzing data from three independent samples collected at different time points, rather than following the same individuals over time. As such the study investigates population-level changes over time. These nationally representative surveys provide comprehensive information about the German workforce and are carried out periodically^17–19^. We used all data from the three most recent surveys, with 2018 being the most recent wave, because the last three surveys used similar sample frames and operationalization of variables and were thus comparable over time. The surveys employed a random-digital-dialing sampling procedure, including both landline and mobile phone numbers. Computer-assisted telephone interviews were conducted, lasting approximately 40 min on average. The interviews covered a wide range of topics, including sociodemographic characteristics, work activities, working conditions, and health.

Participants aged 15 and over working at least 10 h per week are surveyed. All procedures were in accordance with the ethical standards of the institutional research committee and with the 1964 Helsinki declaration and its later amendments. Informed consent was obtained from all subjects and/or their legal guardians. Adolescents provided consent themselves, as the survey topics were non-sensitive and non-intrusive. The BAuA ethics committee granted approval for the surveys, with the most recent approval being EK007_2017 on January 9, 2017. After omitting participants with missing values listwise (n = 6301, approximately 10% of the original sample size), a final sample of N = 53,747 participants resulted.

### Measures

Self-rated health was measured in the BIBB/BAuA Employment Surveys (2006, 2012 and 2018) using a single item, asking participants to rate their overall health on a 5-point scale (excellent, very good, good, fair, or poor). This single-item measure follows international standards and has been extensively validated in population health research, demonstrating strong predictive validity for mortality and functional status across diverse populations^20^. Research has also consistently shown that this measure captures multiple dimensions of health status including physical, mental, and social well-being components. The measure has been widely used in major epidemiological studies and population surveys worldwide due to its ability to economically provide reliable population-level health assessments^21^. Responses were dichotomized into "at least good" (excellent, very good, or good) versus “not good” (fair or poor) health.

Work activities and working conditions were measured using a series of items assessing the frequency of different tasks and exposures. For the work activity items, participants could choose to respond with “frequently” (coded as 2), “sometimes” (coded as 1), or “never” (coded as 0). Physical work activities were measured with 6 items asking about the frequency of manufacturing, measuring, controlling, repairing, transporting, and cleaning work tasks (Cronbach’s α = 0.71). Cognitive work activities were assessed with 6 items on the frequency of planning, researching, educating, searching for information, advising, and computer use work tasks (Cronbach’s α = 0.71). For the working conditions items, a four-point scale was used inquiring about the frequency with which the participants had to work under certain conditions, with the following response options: “frequently” (coded as 3), “sometimes” (coded as 2), “seldom” (coded as 1), or “never” (coded as 0). Ergonomic conditions were operationalized as the mean of 2 items: lifting heavy loads and working in awkward postures (Cronbach’s α = 0.72). Environmental conditions were operationalized as the mean of 6 items: exposure to smoke/dust/fumes, unfavorable climate conditions, oil/grease/dirt, poor lighting, noise, and microorganisms (Cronbach’s α = 0.76). Work intensity was operationalized as the mean of 4 items: time pressure, working very quickly, working to the limits of one’s capacity, and multitasking (Cronbach’s α = 0.67). Work control was operationalized as the mean of 4 items (Cronbach’s α = 0.44): autonomy in work planning, influence over workload, and two items on access to information (reverse coded). Social support was operationalized as the mean of 2 items: feeling part of a community at work and good cooperation with colleagues (Cronbach’s α = 0.59).

Several covariates were included in the analyses. Age was measured in years. Gender was operationalized as male vs. female. Occupation was categorized into four groups based on the International Standard Classification of Occupations (ISCO) major group codes: high-skilled white-collar (ISCO 1–3, which corresponds to Managers, Professionals, and Technicians and Associate Professionals), low-skilled white-collar (ISCO 4–5, which corresponds to Clerical Support Workers and Services and Sales Workers), high-skilled blue-collar (ISCO 6–7, which corresponds to Skilled Agricultural, Forestry and Fishery Workers and Craft and Related Trades Workers), and low-skilled blue-collar (ISCO 8–9, which corresponds to Plant and Machine Operators and Assemblers and Elementary Occupations). Working hours were measured as the average number of work hours reported per week. Education level was based on school educational attainment and categorized as low (up to the German “Hauptschule”, which is the basic secondary school track typically completed after 9 years of schooling), intermediate (up to the German “Realschule”, which is the intermediate secondary school track typically completed after 10 years of schooling), or high (German “Abitur”, which is the highest secondary school qualification that grants university entrance qualification, typically completed after 12–13 years of schooling, and above).

### Data analysis

First, descriptive statistics of all variables were calculated for each survey year (2006, 2012, and 2018) and for the overall sample. These statistics included means and standard deviations for continuous variables, and percentages for categorical variables. Then, logistic regression analyses were conducted to study trends in self-rated health (SRH) over time. The outcome variable was dichotomized SRH (0 = poor/fair health, 1 = good/very good/excellent health). The main predictor variable was the time period, which was scaled fractionally such that 2006 = 0 and 2018 = 1, allowing the interpretation of its coefficient as the average change in the odds of reporting good health over the study period.

To examine the extent to which the time trends could be explained by work-related factors, two analytical approaches were utilized. First, models of increasing complexity were calculated^22^: Model 1 included only the time period as a predictor. Model 2 added sociodemographic variables: age, gender, working hours, occupation (categorized as white-collar high-skilled, white-collar low-skilled, blue-collar high-skilled, and blue-collar low-skilled), and education level (low, intermediate, high). Model 3 further included work tasks and working conditions: physical work, cognitive work, ergonomic conditions, environmental conditions, work intensity, job control, and social support. For each model, odds ratios (ORs) with 95% confidence intervals were calculated. Changes in the effect size of the time period coefficient between models indicate the degree to which the added variables explain the observed trends in SRH.

Additionally, mediation analyses were conducted to determine whether changes in work activities and conditions significantly explained the relationship between time period and self-rated health. Following the causal mediation approach developed by Imai and colleagues, we estimated mediation effects separately for each potential mediator (physical work tasks, cognitive work tasks, ergonomic conditions, environmental conditions, work intensity, job control, and social support) to isolate their potential contributions to the time trend^23^. For each mediator, we fitted two models: a mediator model predicting the mediator from the time period and covariates (Age, Gender, Working Hours, Occupational Group, Education), and an outcome model predicting self-rated health from both the mediator and time period, including the same covariates. Bootstrapping with 500 simulations was used to obtain confidence intervals and p-values. Results are reported as proportions mediated, which can be interpreted as the percentage of the total time trend in good self-rated health explained by each mediator, as well as the Average Causal Mediated Effect (ACME) representing the absolute magnitude of the indirect effect through each mediator (i.e., the average change in the probability of good self-rated health over time that is due to the mediator). All analyses were weighted using the survey weights provided to ensure better representativeness. In general, this statistical approach can be expected to be very robust given the substantial sample size and thus statistical power: Our sample size (n = 53,747) far exceeds the recommended minimum of 10–15 cases per predictor variable for regression analysis. Also, multicollinearity diagnostics revealed no problematic relationships among predictor variables, with variance inflation factors < 2.0 (and thus being far below the conventional cutoff of < 10), further supporting our approach. All analyses were performed using R statistical software.

## Results

As depicted in Table 1, participants were on average 43.75 years old (SD = 11.03), with 51.3% being female. The majority of participants belonged to the white-collar high-skilled occupational group (53.6%), followed by white-collar low-skilled (21.6%), blue-collar high-skilled (13.3%), and blue-collar low-skilled (11.5%). Overall, 87.7% of participants reported good self-rated health. Self-rated health changed over time on a descriptive level, with the percentage of participants reporting good health decreasing from 90.9% in 2006 to 86.6% in 2018. Concurrently, several socio-demographic and work characteristics changed, including age (from 41.42 years in 2006 to 44.73 years in 2018), female participation (from 50.5% to 48.2%), and occupational group composition (white-collar high-skilled increasing from 50.3% to 60.8%). Changes were also observed in physical activities (1.71 to 1.67), cognitive activities (2.08 to 2.21), ergonomic conditions (0.97 to 0.92), work intensity (2.11 to 2.05), and work control (1.91 to 1.83). All changes over time had small to moderate effect sizes, as indicated by the SMD values being less than 0.5, with education showing the largest change (SMD = 0.308).

**Table 1:** Self-rated health, work characteristics and sociodemographic characteristics.

	Overall	Stratified by Year			SMD
		2006	2012	2018	
N	53,747	18,005	17,848	17,894	
Self-rated Health (% good)	87.7	90.9	85.2	86.6	0.119
Age (mean (SD))	43.75 (11.03)	41.42 (10.40)	45.43 (10.63)	44.73 (11.88)	0.247
Gender (% female)	51.3	50.5	54.0	48.2	0.077
Working Hours (mean (SD))	38.07 (12.06)	38.19 (12.72)	37.95 (11.94)	38.08 (11.20)	0.014
Occupational Group (%)					0.148
WC–HS	53.6	50.3	52.0	60.8	
WC–LS	21.6	22.7	23.0	18.1	
BC–HS	13.3	14.8	13.5	10.9	
BC–LS	11.5	12.3	11.5	10.2	
Education (%)					0.308
Low	23.8	27.6	25.6	15.3	
Intermediate	39.2	40.7	42.4	32.4	
High	37.0	31.7	32.0	52.3	
Physical Activities (mean (SD))	1.70 (0.51)	1.71 (0.51)	1.70 (0.51)	1.67 (0.51)	0.056
Cognitive Activities (mean (SD))	2.13 (0.49)	2.08 (0.50)	2.12 (0.49)	2.21 (0.47)	0.183
Ergonomic Conditions (mean (SD))	0.97 (1.00)	0.97 (0.99)	1.01 (1.00)	0.92 (1.01)	0.064
Environmental Conditions (mean (SD))	0.76 (0.73)	0.75 (0.73)	0.75 (0.72)	0.77 (0.75)	0.012
Work Intensity (mean (SD))	2.08 (0.64)	2.11 (0.63)	2.08 (0.65)	2.05 (0.65)	0.062
Work Control (mean (SD))	1.90 (0.59)	1.91 (0.59)	1.92 (0.59)	1.83 (0.60)	0.095
Work Support (mean (SD))	2.78 (0.48)	2.77 (0.48)	2.79 (0.47)	2.79 (0.48)	0.027

N, sample size; SD, Standard Deviation; SMD, Standardized (Mean) Difference; WC–HS, White-Collar High Skilled; WC–LS, White-Collar Low Skilled; BC–HS, Blue-Collar High Skilled; BC–LS, Blue-Collar Low Skilled.

### Time trends in SRH

As depicted in Table 2, self-rated health changed significantly across time periods in the regression analyses. In the unadjusted model, the odds of reporting good health decreased by 36% across the time period (OR = 0.64, 95% CI [0.60 0.69]). After adjusting for socio-demographic factors in Model 2, the effect of time period remained similar (OR = 0.63, 95% CI [0.58, 0.68]). However, in the fully adjusted Model 3, which included work-related factors, the negative effect of time period on self-rated health slightly attenuated (OR = 0.67, 95% CI [0.62, 0.73]). Regarding the other predictors of SRH in the full model, age was negatively associated with good health (OR = 0.96, 95% CI [0.95, 0.96]), while being female was associated with lower odds of good health (OR = 0.63, 95% CI [0.591 0.68]). Education showed a positive gradient, with higher education levels associated with better self-rated health (Intermediate: OR = 1.33, 95% CI [1.23, 1.43]; High: OR = 1.69, 95% CI [1.54, 1.85]). Among work-related factors, work control (OR = 1.61, 95% CI [1.53, 1.69]) and work support (OR = 1.81, 95% CI [1.72, 1.90]) showed strong positive associations with SRH, while work intensity (OR = 0.62, 95% CI [0.59, 0.65]) and environmental conditions (OR = 0.74, 95% CI [0.70, 0.78]) demonstrated substantial negative associations.

**Table 2:** Logistic regression results predicting self-rated health over time.

Variable	OR	95%-CI	*p*	XXX	OR	95%-CI	*p*	XXX	OR	95%-CI	*p*
Time period	0.64	[0.60; 0.69]	< .001		0.63	[0.58; 0.68]	< .001		0.67	[0.62; 0.73]	< .001
Age					0.96	[0.96; 0.97]	< .001		0.96	[0.95; 0.96]	< .001
Gender					0.60	[0.56; 0.64]	< .001		0.63	[0.59; 0.68]	< .001
Working hours					0.99	[0.99; 1.00]	< .001		1.01	[1.00; 1.01]	< .001
Occupation											
WC–HS (Ref.)					–	–	–		–	–	–
WC–LS					0.90	[0.83; 0.97]	.006		0.98	[0.91; 1.07]	.659
BC–HS					0.73	[0.66; 0.80]	< .001		1.07	[0.95; 1.19]	.273
BC–LS					0.58	[0.53; 0.63]	< .001		0.83	[0.74; 0.92]	< .001
Education											
Low (Ref.)					–	–	–		–	–	–
Intermediate					1.35	[1.26; 1.45]	< .001		1.33	[1.23; 1.43]	< .001
High					1.94	[1.79; 2.11]	< .001		1.69	[1.54; 1.85]	< .001
Physical activities									1.09	[1.01; 1.18]	.023
Cognitive activities									1.14	[1.06; 1.24]	< .001
Ergonomic conditions									0.91	[0.87; 0.94]	< .001
Environmental conditions									0.74	[0.70; 0.78]	< .001
Work intensity									0.62	[0.59; 0.65]	< .001
Work control									1.61	[1.53; 1.69]	< .001
Work support									1.81	[1.72; 1.90]	< .001

OR, odds ratio; CI, confidence interval; WC–HS, white collar, high skilled; WC–LS, white collar, low skilled; BC–HS, blue collar, high skilled; BC–LS, blue collar, low skilled.

Finally, mediation analyses revealed that work control and environmental conditions were the strongest mediators, explaining 19% (ACME = − 0.009, *p* < 0.001) and 14% (ACME = − 0.007, *p* < 0.001) of the time trend (total effect between time period and SRH, representing a decline of about 5% in SRH over time), respectively. Ergonomically hazardous conditions explained 8% (ACME = − 0.004, *p* < 0.001) of the effect, while physical work tasks accounted for 2.3% (ACME = − 0.001, *p* < 0.001). Changes in work intensity showed a negative mediation effect (− 11%, ACME = 0.005, *p* < 0.001), suggesting that the declining work intensity partially protected against further decline in self-rated health. Cognitive work tasks showed a minimal negative mediation effect (− 1%, ACME = 0.001, *p* < 0.001), and workplace social support showed no significant mediation (ACME = 0.000, *p* = 0.320).

## Discussion

We asked how self-rated health had changed among workers, and whether these trends could be explained by changing work characteristics. We found that self-rated health significantly deteriorated among workers between 2006 and 2018. This worsening trend occurred alongside notable demographic shifts in the workforce, including aging and more educated workers. We also documented various changes in working conditions and activities over time: Work became generally less physically but more cognitively and psychosocially demanding. When we accounted for these changes in our models, they partially explained the declining trend in worker self-rated health.

Our findings both corroborate and extend previous research. The observed decline in self-rated health aligns with studies documenting health deteriorations among middle-aged and older adults^14–16^. For instance, Beller and colleagues, using longitudinal SHARE data from Germany, reported increased movement-related and general activity limitations among the working population^15^. Our study thus reinforces these findings on declining worker health. However, we advance beyond most existing research by systematically investigating whether changes in the work environment, such as shifts in job activities and psychosocial conditions, could explain trends in worker self-rated health. While these work factors partially accounted for the trends, they did only explain the overall worsening pattern observed in our study to a moderate degree. This suggests that determinants outside the immediate work environment and/or substantial subgroup differential effects likely also play a significant role in the declining health trends among workers.

### Explanations

The deterioration in self-rated health among German workers could partially be explained by work-related conditions in our study, with work control and hazardous environmental conditions emerging as particularly important mediators for explaining the negative health trends. This suggests that declines in job control and increases in occupational environmental hazards may be current contributing trends impacting population health^24–26^. Some of these work-related factors showed significant mediating effects despite relatively small descriptive changes over time, which might be due to their strong relationship with health outcomes and complex interrelationships with other variables, including the sociodemographic changes over time, when accounting for the covariates. In support of this possibility, work control and environmental working conditions were also most strongly associated with SRH in the current analysis. However, since the work-related factors only partially explained the observed health trends, future studies should investigate additional mechanisms as to why SRH deteriorated over time.

There are several possible additional explanations for the observed deterioration in self-rated health among workers in Germany beyond the studies changes in job activities and working conditions. One potential explanation is the increasing prevalence of chronic diseases and mental health issues among the working population. For example, several studies have shown that the prevalence of chronic conditions such as diabetes, cardiovascular diseases, and musculoskeletal disorders has been on the rise in recent years^27–31^. These health issues can significantly impact an individual’s overall well-being and self-rated health, regardless of changes in job activities and working conditions.

Furthermore, the changes in job activities and working conditions may not fully capture the complexity of the modern work environment. For example, the increasing use of technology and digitalization in the workplace may have both positive and negative effects on worker health. While technology can reduce physical demands and improve efficiency, it can also lead to increased work intensity, information overload, and technostress, which may negatively impact self-rated health^32^. In conclusion, the deterioration in self-rated health among workers in Germany is likely the result of a complex interplay of factors, including but also going beyond changes in work activities and working conditions.

Another possible explanation is the influence of factors outside the workplace, such as lifestyle behaviors^33^. Unhealthy lifestyle behaviors, including physical inactivity, poor diet, smoking, and excessive alcohol consumption, have been associated with poorer self-rated health^34,35^. In recent years, there has been a growing concern about the impact of a sedentary lifestyle on health and well-being^36^. This may have become more prevalent in recent years, contributing to the observed trends in self-rated health^5,37^. The influence of these lifestyle factors on the health trends we observed could be multifaceted and substantial. Germany, like many developed countries, has experienced rising rates of obesity, type 2 diabetes, and metabolic syndrome during the period covered by our study^29^. These conditions are closely linked to lifestyle factors such as diet quality, physical activity levels, and stress management, all of which could have deteriorated independent of workplace changes. Additionally, the increasing prevalence of mental health issues, including depression and anxiety disorders, may reflect broader social pressures, economic uncertainty, and changing social support structures that extend beyond the immediate work environment^38^. Furthermore, lifestyle behaviors may interact with work characteristics in complex ways that our analysis could not capture. For example, workers experiencing high job demands or low job control may be more likely to engage in unhealthy coping behaviors such as smoking, excessive alcohol consumption, or emotional eating. Conversely, demanding work schedules may limit opportunities for physical activity, meal planning, or adequate sleep, creating indirect pathways through which work affects health via lifestyle mediators. Lastly, future studies are needed to examine this topic further.

An important finding that warrants specific discussion is also the seemingly non-linear pattern in self-rated health across our study period. Rather than showing consistent decline from 2006 to 2018, our data reveal health deterioration from 2006 to 2012 followed by a slight but not complete improvement from 2012 to 2018. This fluctuation could reflect economic cycles, policy changes, or other temporal factors that influence health independently of gradual changes in working conditions. Future research should employ more frequent measurement points (annual or biannual surveys) to better understand the timing and drivers of such health oscillations. Such research would also help distinguish between temporary fluctuations and genuine long-term trends in worker health. One possibility might be that the pattern observed in our study reflects the global financial crisis that was especially visible in 2012 and the subsequent economic recovery periods, thus suggesting macroeconomic associations with worker health. However, this hypothesis must specifically be tested in future research.

The shift towards higher-skilled occupations provides additional context for interpreting our main findings. The occupational health gradients observed in our study also align with established literature showing better health outcomes among higher-skilled occupations^39^. The fact that self-rated health declined over time despite the workforce becoming more concentrated in typically healthier occupational categories (white-collar high-skilled positions) suggests that the deterioration we observed is not an artifact of changing occupational composition. Instead, it indicates that health declines occurred within occupational groups, potentially due to changing work characteristics and conditions that affected workers across different occupational strata. This pattern strengthens our argument that changes in job activities and working conditions, rather than simple demographic shifts, are important contributors to the observed health trends. The mediation analyses support this interpretation by showing that specific work-related factors, particularly work control and environmental conditions, help explain the time trends.

### Implications

From a theoretical perspective, these results go against the compression of morbidity hypothesis. The compression of morbidity hypothesis states that improvements in working and living conditions and healthcare would lead to better health outcomes and compressed periods of morbidity^40,41^. The observed deterioration in self-rated health directly contradicts the expected compression of morbidity. Instead, the results support the expansion of morbidity hypothesis among the working population^42^. The expansion of morbidity hypothesis suggests that medical advances and improved living conditions primarily postpone death without necessarily preventing the onset of chronic conditions or functional limitations. Our findings align with this theory, showing that workers are experiencing health problems earlier in their careers despite having better education and less physically demanding jobs.

From a practical perspective, the study’s results underscore the importance for interventions and policies aimed at promoting worker health and well-being. Self-rated health at work has been shown to be especially relevant for predicting crucial occupational outcomes, including work disability and early retirement intentions^43,44^. Such interventions may include measures to address the psychosocial as well as physical aspects of work, such as fostering a supportive work environment^45^. Furthermore, efforts should be made to support workers with poor self-rated health and chronic health conditions^46^. However, future studies are also needed to further elaborate the reasons for the decline in SRH.

### Limitations

The current study has several limitations that should be considered when interpreting the results. First, regarding our health outcome measure, while self-rated health is a well-established and validated construct in population health research with strong predictive validity, the use of a single-item measure may not capture the full complexity of health status that multi-dimensional health assessment tools could provide^20^. Future research might benefit from incorporating more comprehensive health measures that assess specific domains of physical and mental health separately, particularly in mental health contexts where more nuanced health assessments may be warranted. One similar major limitation is the reliance on self-reported measures for both self-rated health and job activities and working conditions. Self-reported data may be subject to various biases, such as recall bias and social desirability bias, which can affect the accuracy and reliability of the findings^47^.

Another significant limitation of this study is that we did not formally test for measurement invariance of the self-rated health measure across the three time points. Measurement invariance testing is crucial for establishing whether observed differences over time reflect true changes in the underlying construct rather than changes in how respondents interpret or respond to the measure. While the single-item self-rated health measure was administered identically across all survey waves using the same five-point scale and standardized protocols, cultural changes in health expectations, increased health awareness, or shifts in what constitutes “good” health could potentially contribute to apparent declines. For example, effects like the Tocqueville paradox could also partially explain the findings, whereby improvements in social or working conditions can paradoxically lead to increased expectations and greater dissatisfaction, potentially resulting in worse health perceptions despite objective improvements^48^. The substantial magnitude of the observed effects, however, suggests that genuine health changes likely contribute to our findings in our view, but formal measurement invariance testing would be needed to quantify the relative contributions of true change versus measurement artifacts.

Moreover, individuals with poor health status may be systematically underrepresented in the study sample, as severe health complaints could impede survey participation through various mechanisms^49^. Self-rated health is a subjective measure that may be influenced by factors beyond the individual’s actual health status, such as cultural norms and personal expectations^20^. Another limitation is the relatively small timeframe covered by the study, with data from only three time points spanning 12 years. While this allows for the examination of comparatively shorter trends, it may not fully capture long-term changes in self-rated health and working conditions. Moreover, the time intervals between the surveys (6 years) may not be optimal for detecting more subtle or gradual changes in the variables of interest. The study’s design also limits the ability to establish causal relationships between the investigated variables. As an observational study using cross-sectional data, it is not possible to determine the directionality of the associations or rule out the influence of potential confounding factors. Finally, the study’s focus on the working population in Germany may limit the generalizability of the findings to other countries or populations. The specific cultural, social, and economic context of Germany may influence the observed trends and associations. As such, future studies using samples from further countries are needed.

## Conclusions

In conclusion, this study reveals a significant decline in self-rated health among German workers between 2006, 2012 and 2018, which could be partially explained by changes in work activities and working conditions. Work control and environmental conditions emerged as important mediators, though they explained only a moderate portion of the observed trends. These findings challenge the compression of morbidity hypothesis and suggest that factors beyond the immediate work environment may be further contributing to declining worker health. Future research should investigate additional mechanisms driving the heath deteriorations and investigate the generalizability of findings. The results underscore the need for comprehensive workplace health promotion strategies that address both traditional occupational hazards and emerging psychosocial challenges in the modern work environment.

## Data Availability

The data that support the findings of this study are available from the Federal Institute for Vocational Education and Training (BIBB) at https://www.bibb.de/en/2815.php.
